# Subclinical Diastolic Indices in Children with Type 1 Diabetes: A Cross-Sectional Comparative Study from Northern Iran

**DOI:** 10.5812/ijem-164686

**Published:** 2026-04-24

**Authors:** Atoosa Rezvani, Hamidreza Mirzaei Ilali, Hadis Ebrahimzade, Hemmat Gholinia Ahangar, Faeze Aghajanpour, Morteza Alijanpour

**Affiliations:** 1 Student Research Committee, Babol University of Medical Sciences, Babol, Iran; 2 Clinical Research Development Unit of Amirkola Children's Hospital, Babol University of Medical Sciences, Babol, Iran; 3 Health Research Institute, Babol University of Medical Sciences, Babol, Iran; 4 Non-Communicable Pediatric Diseases Research Center, Health Research Institute, Babol University of Medical Sciences, Babol, Iran

**Keywords:** Cardiovascular Disease, Child, Echocardiography, Iran, Type 1 Diabetes Mellitus

## Abstract

**Background:**

Type 1 diabetes mellitus (T_1_DM) is common and can cause cardiac complications. Subclinical myocardial involvement, particularly impaired diastolic function, is a concern in pediatric patients with T_1_DM.

**Objectives:**

This study aimed to assess cardiac function, with a particular focus on diastolic indices, in children with T_1_DM.

**Methods:**

In this cross-sectional comparative study, children aged 6 - 12 years were divided into two groups: a case group with T_1_DM and a control group of healthy children. Participants were recruited in 2023 from the Endocrinology Clinic at Amirkola Children's Hospital, Babol, Iran. All children underwent clinical evaluation, pubertal status assessment using the Marshall-Tanner criteria, electrocardiography, and echocardiography.

**Results:**

A total of 150 children were included, comprising 50 children with T_1_DM and 100 controls. Overall, 52% of the participants were boys, and the mean age was 9.28 ± 1.85 years. Late diastolic tissue velocity (A') was significantly higher in the T_1_DM group (P = 0.02), suggesting subclinical diastolic impairment. However, most other cardiac parameters, including all systolic indices, ejection fraction, fractional shortening, and electrocardiographic findings, were comparable between the groups (all P > 0.4). The T_1_DM group also had higher mean weight, height, body mass index, and body mass index percentiles (all P < 0.05).

**Conclusions:**

Children with T_1_DM exhibited subtle diastolic dysfunction, reflected by elevated A', whereas systolic function and other cardiac parameters were comparable with those in controls. Given the cross-sectional design, limited sample size, and potential confounding by body mass index and selection bias, these findings should be interpreted cautiously. The standardized single-center methodology supports internal validity; however, multicenter longitudinal studies with larger, z-score-adjusted samples are needed to confirm these results and clarify their clinical implications.

## 1. Background

Diabetes mellitus (DM) comprises a group of metabolic diseases characterized by hyperglycemia resulting from defects in insulin secretion, insulin action, or both (1). DM is a major global health, medical, social, and economic problem and is divided into 2 main forms: type 1 diabetes mellitus (T_1_DM), or insulin-dependent diabetes, and type 2 diabetes mellitus (T2DM), or non-insulin-dependent diabetes (2). The global incidence of T_1_DM has increased significantly, and T_1_DM is the most common chronic disease in childhood (3-5). The incidence of childhood T_1_DM varies by geographic region, age, sex, family history, and ethnicity, with specific incidence rates reported in regions such as Finland, Sardinia, and the United States (6-8). Although most autoimmune diseases are more common in females than in males, the incidence of T_1_DM in children does not differ significantly between boys and girls (9). The diagnostic criteria for DM include fasting blood sugar ≥ 126 mg/dL or an oral glucose tolerance test ≥ 200 mg/dL.

The age at manifestation of T_1_DM has 2 peaks: 1 at 4 - 6 years and another around the onset of puberty, at 10 - 14 years (10). Overall, 45% of affected children develop symptoms before 10 years of age (11). Seasonal variation also appears to exist, with more cases diagnosed in autumn and winter. For example, in a study by Alijanpour et al. conducted from 2011 to 2017, the incidence of T_1_DM in pediatric patients increased significantly during cold seasons, especially winter. In the age group of 5 - 9 years, the prevalence was higher in cold seasons than in other groups; however, the distribution of birth seasons was similar across groups (12).

Several factors, including urbanization, an unhealthy diet, a sedentary lifestyle, and lack of exercise, contribute to the development of diabetes (13). In addition, increasing mean age and population aging are important risk factors for diabetes, as its incidence increases with age (14). In genetically predisposed individuals, exposure to 1 or more environmental factors triggers an immune response that ultimately leads to destruction of the insulin-secreting beta cells of the pancreas (7). Environmental factors that increase the risk of T_1_DM include viral infections, especially respiratory and enteroviral infections; nutritional factors, such as cow's milk consumption during infancy, obesity, and vitamin D deficiency; perinatal factors, including advanced maternal age and a maternal history of preeclampsia; and neonatal factors, including jaundice, prematurity, and high birth weight (15, 16).

Diabetes not only reduces quality of life and life expectancy but is also a major cause of microvascular and macrovascular complications, leading to blindness, kidney failure, myocardial infarction, stroke, and limb amputation (17, 18).

**Figure 1.**
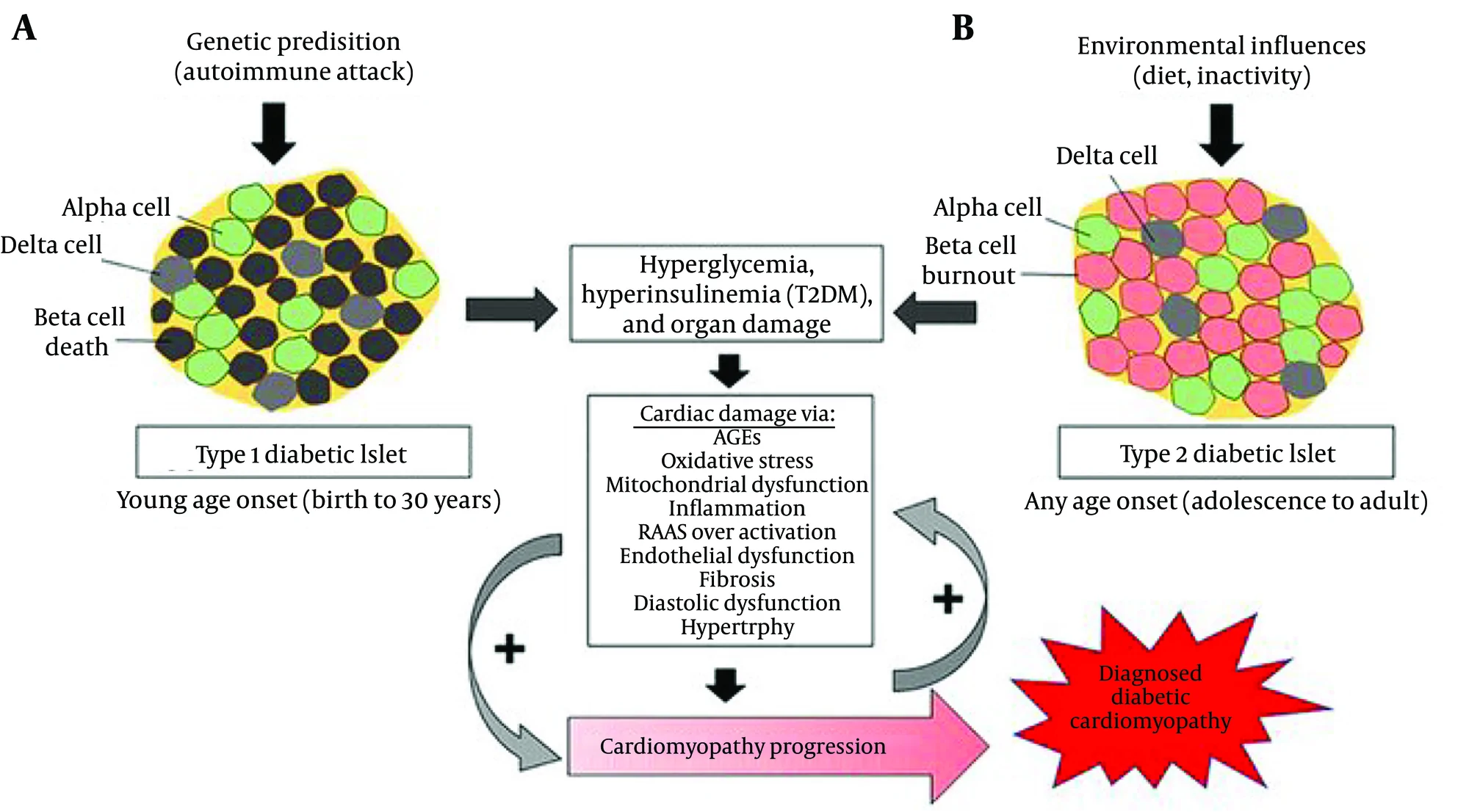
Comparison of the distinct routes to diabetic cardiomyopathy. Type 1 diabetes mellitus develops primarily from immune-mediated hyperglycemia (A), whereas type 2 diabetes mellitus progresses through insulin resistance and metabolic dysfunction (B), with both pathways ultimately culminating in cardiac impairment.

Diastolic dysfunction secondary to T_1_DM is influenced by disease duration, age at onset, and management. The myocardial impact of T_1_DM differs from that of T2DM, complicating the definition of diabetic cardiomyopathy, as T_1_DM is often associated with hyperglycemia and myocardial fibrosis in a younger cohort. The mechanism of T_1_DM-associated diastolic dysfunction is illustrated in Figure 1 (19).

Cardiovascular disease remains the most common cause of premature death in patients with T_1_DM (20, 21), with a 2- to 4-fold increased risk compared with the general population (22). The spectrum of diabetic heart disease includes progression from a normal heart to left ventricular diastolic and systolic dysfunction, followed by overt echocardiographic evidence of left ventricular dysfunction and, ultimately, symptomatic heart failure (23). However, many patients remain asymptomatic (24).

## 2. Objectives

Given that diabetes is a common disease with many complications, that cardiac complications are rare but severe, and that no studies have been conducted in northern Iran, the current study aimed to compare cardiac function between children with and without T_1_DM.

## 3. Methods

### 3.1. Study Design

**Figure 2.**
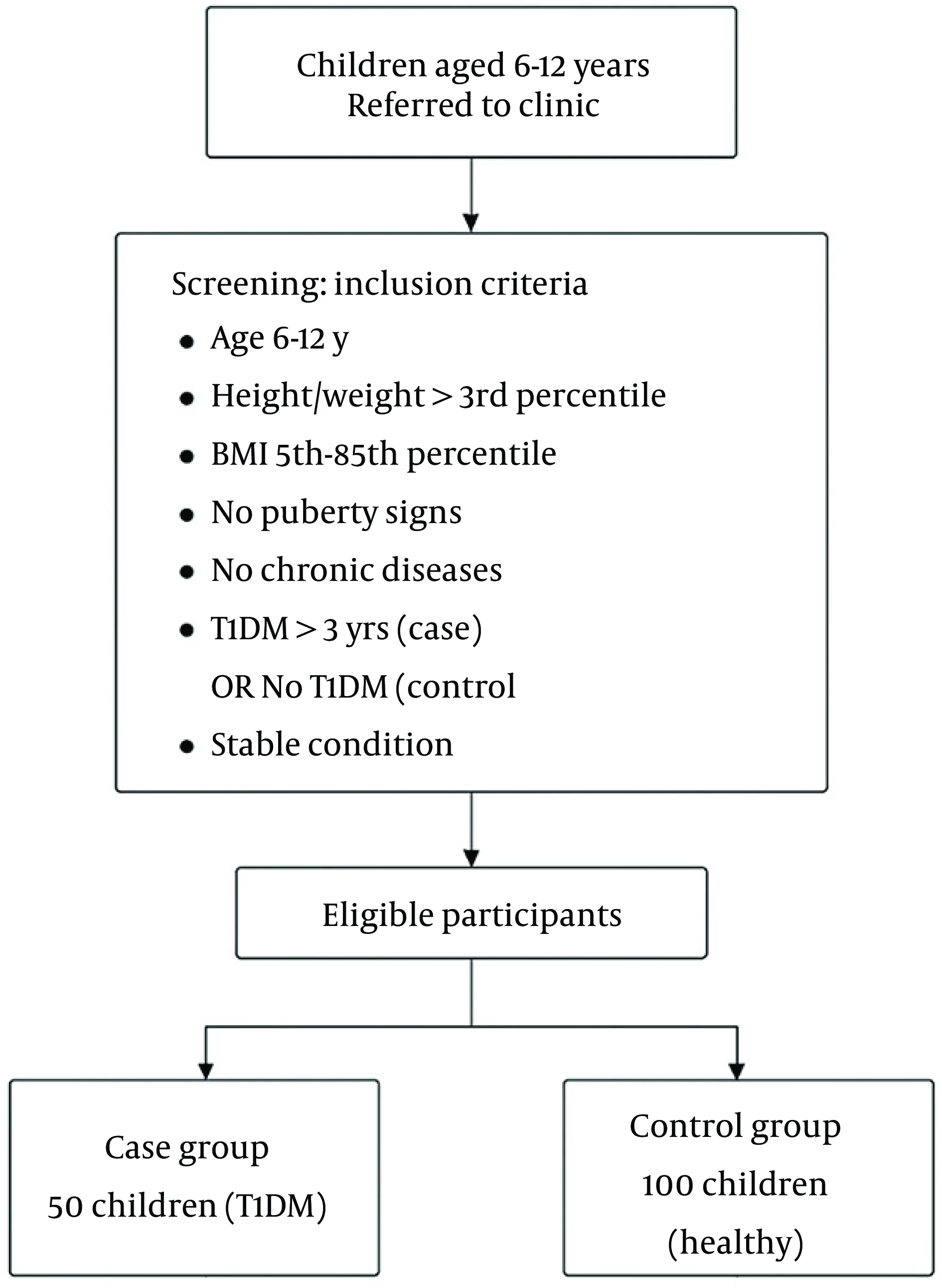
Flowchart of participant selection and study procedure.

This cross-sectional comparative study was conducted in 2023 among children aged 6 - 12 years with and without T_1_DM who were referred to the Endocrinology and Cardiology Clinics of Amirkola Children's Hospital, Babol, Iran. The study sample comprised 50 children with T_1_DM as the case group and 100 healthy children as the control group, selected using convenience sampling (Figure 2).

The inclusion criteria were as follows: Age 6 - 12 years; height and weight > 3rd percentile for growth; normal body mass index (BMI; 5th - 85th percentile); no signs of puberty; no underlying chronic diseases (cardiac, pulmonary, renal, gastrointestinal, etc) in either the case or control group; presence of T_1_DM for at least 3 years in the case group or absence of T_1_DM in the control group; and a stable clinical condition, defined as no recent acute infection or diabetic ketoacidosis.

The exclusion criteria were noncompliance and identification of previously undetected congenital heart disease during the study.

The sample size was calculated based on the myocardial performance index (Tei index) reported by Kim et al. (25), which was 0.4 in the control group and 0.5 in the case group. Based on the sample size formula, 50 children were calculated for the case group and 100 individuals for the control group.

$$n=\frac{{\left(Z_{1-\frac{\alpha }{2}}+Z_{1-\beta }\right)}^{2}(S_{1}^{2}+S_{2}^{2})}{d^{2}}=50$$

- α = 0.05, β = 0.20, S_1_ = S_2_ = 0.1, d = 0.05.

- MDC = 0.05, mean of the control group = 0.4, SD of the control group = 0.1; n = 38.

- Mean of the case group = 0.5, SD of the case group = 0.1; n = 47.

- Pooled SD = 0.1, alpha = 0.05, power = 80%, effect size = 0.76.

In the present study, children underwent a comprehensive general and pubertal examination by a pediatric endocrinologist, assisted by a pediatric resident. Children aged 6 - 12 years without signs of puberty were included. As shown in Table 1 (26-30), because of the potential confounding effects of pubertal stages on cardiovascular structure and function, participants with clinical signs of puberty were excluded.

**Table 1.** Effects of Puberty on Cardiac Structure and Function

Pubertal Effect	Cardiac/Functional Change
**Increase in lean body mass and growth hormones**	Increase in left ventricular mass, wall thickness, and chamber size
**Sex hormones - testosterone**	Promotes left ventricular hypertrophy and greater chamber dimensions in boys
**Sex hormones - estrogen**	Vasodilatory and protective vascular effects, with lower blood pressure trajectories in girls
**Pubertal growth spurt**	Altered myocardial strain and twist/untwist; diastolic function is sensitive
**Autonomic and neurohormonal shifts**	Decreased vagal tone and increased sympathetic drive; predisposition to syncope/postural orthostatic tachycardia syndrome
**Blood pressure trajectories**	Increased blood pressure and arterial stiffness, especially in boys
**Early puberty timing**	Higher adult left ventricular mass index, increased wall thickness, and increased risk of hypertension
**Lifestyle and environmental factors**	Obesity, inactivity, and endocrine disruptors worsen adverse remodeling

The case group included children with T_1_DM who had been diagnosed at least 3 years earlier. They were divided into 2 groups based on HbA1c levels: 6.8 - 8 as good control and > 8 as poor control. The control group consisted of healthy children aged 6 - 12 years without diabetes and without signs of puberty who were referred to the Endocrinology Clinic for growth monitoring.

All enrolled children were referred to the Cardiology Clinic for cardiac examination. Initially, an electrocardiogram (ECG) was performed for all participants by trained clinic nursing staff. Subsequently, transthoracic echocardiography was performed for all children using a MEDISON echocardiography device (Korea). To maximize operator consistency, transthoracic echocardiography for each participant was performed exclusively by a single experienced pediatric cardiologist. This operator was strictly blinded to group assignment (diabetic vs nondiabetic) to minimize acquisition and interpretation bias. Echocardiographic data, including chamber dimensions and functional parameters, were collected, and the resulting measurements were compared between the diabetic and control groups.

### 3.2. Echocardiographic Acquisition

Transthoracic echocardiography was performed with the child in the left lateral decubitus position using standard pediatric imaging windows. Images were acquired from parasternal long-axis, parasternal short-axis, apical 4-chamber, and apical 5-chamber views in accordance with the American Society of Echocardiography pediatric guidelines. Doppler and tissue Doppler measurements were averaged over 3 consecutive cardiac cycles in sinus rhythm to minimize beat-to-beat variability. Tissue Doppler velocities, including early diastolic annular peak velocity (E') and late diastolic tissue velocity (A'), were obtained from the septal mitral annulus with careful alignment of the Doppler beam with myocardial motion.

### 3.3. Quality Control

All echocardiographic examinations were performed and analyzed by a single experienced pediatric cardiologist who was blinded to group allocation, ensuring uniform acquisition and interpretation. Given the single-operator design, interobserver variability was not assessed; however, this approach minimized measurement variability across the study population.

A standard 12-lead ECG was obtained for every participant. The following parameters were measured: heart rate, PR interval, QRS duration, QT interval corrected for heart rate using the Bazett formula (QTc), QRS axis, and the presence of arrhythmias, conduction abnormalities, or ST-T wave changes. All ECGs were interpreted by a pediatric cardiologist who was blinded to group assignment.

### 3.4. Indices for Cardiac Performance Assessment

Various indices are used in echocardiography to assess cardiac function and detect abnormalities in patients with cardiovascular disease (Table 2) (31-35). These include ejection fraction (EF), fractional shortening (FS), early and late diastolic filling velocities (E and A), E/A ratio, tricuspid annular plane systolic excursion (TAPSE), Tei index, tissue Doppler early diastolic annular peak velocity (E'), E/E' ratio, late diastolic tissue velocity (A'), E'/A' ratio, and deceleration time (DT). Accurate evaluation of these indices may aid in diagnosis and appropriate treatment, as described below (36-39).

**Table 2.** Normal Pediatric Echocardiographic Function Parameters ^a^

Parameters	Normal Pediatric Values	Notes
**Left ventricular ejection fraction (Simpson or M-mode)**	≥55% normal; 50% - 54% borderline; < 50% abnormal	Higher values are common in infants
**Fractional shortening**	28% - 45% normal; < 25% abnormal	Depends on preload/afterload
**Left ventricular dP/dt from mitral regurgitation jet**	> 1000 mm Hg/s normal	Load-dependent
**Tricuspid annular plane systolic excursion**	> 10 mm in infants; > 15 mm in adolescents	Right ventricular systolic function
**Right ventricular S' (tissue Doppler imaging, tricuspid annulus)**	> 10 cm/s in children/adolescents	Right ventricular longitudinal systolic function
**Mitral inflow E/A ratio**	Infants: < 1; children/adolescents: > 1	Age-dependent
**Mitral E deceleration time**	140 - 220 ms	Age- and heart rate-dependent; shorter in infants
**Tissue Doppler e' (mitral annulus)**	Septal: > 8 cm/s; lateral: > 10 cm/s	Decreases with age
**E/e' ratio**	< 8 normal filling pressures; > 14 suggests increased filling pressure	-
**Isovolumic relaxation time**	Approximately 50 - 80 ms	Age-dependent; shorter in infants
**Pulmonary vein S/D ratio**	Approximately 1.0 - 1.2 in children	Assesses left atrial filling
**Left atrial volume index**	< 28 mL/m^2^	Z score-adjusted in pediatrics
**Right ventricular fractional area change**	≥ 35%	Index of right ventricular systolic function
**Left ventricular global longitudinal strain**	Approximately -18% to -22% normal	Less validated in infants
**Myocardial performance index (Tei index)**	Left ventricular myocardial performance index: approximately 0.35 - 0.45 normal; right ventricular myocardial performance index: approximately 0.30 - 0.40 normal	Higher values indicate worse combined systolic/diastolic performance and are relatively load-independent

^a^ Abbreviations: LVEF: left ventricular ejection fraction; FS: fractional shortening; LV dP/dt: left ventricular pressure rate of change; TAPSE: tricuspid annular plane systolic excursion; RV S′: right ventricular systolic velocity; TDI: tissue doppler imaging; E/A ratio: mitral inflow peak velocity ratio; DT: deceleration time; e′: early diastolic velocity; E/e′ ratio: early mitral inflow to early diastolic annulus velocity ratio; IVRT: isovolumic relaxation time; PV S/D ratio: pulmonary vein systolic/diastolic ratio; LAVI: Left Atrial Volume Index; RV FAC: right ventricular fractional area change; GLS: global longitudinal strain; MPI: Myocardial Performance Index (Tei Index).

Abnormal echocardiographic values were defined using age-appropriate pediatric reference standards. An E/A ratio < 1.0 or > 2.0 was considered abnormal for children aged 6 - 12 years. A left ventricular Tei index > 0.45 was classified as abnormal. Tissue Doppler A' velocity was interpreted using pediatric normative data and analyzed as a continuous variable because no validated categorical cutoff for abnormal A' exists in children.

Based on prior pediatric echocardiographic studies and guideline recommendations emphasizing the sensitivity of tissue Doppler-derived indices for detecting early myocardial dysfunction, A' was predefined as the primary echocardiographic endpoint of this study. Other systolic and diastolic parameters, including E', E/E' ratio, Tei index, E/A ratio, and conventional systolic indices, were analyzed as secondary exploratory outcomes.

### 3.5. Data Analysis

Data were analyzed using SPSS version 26. Data for the studied children were expressed as mean ± SD or as number and percentage. To compare cardiac function between the case and control groups, the chi-square test was used for categorical variables, and the Fisher exact test was applied when the assumptions of the chi-square test were not met. An independent samples t-test was used to compare the means of quantitative variables, including cardiac function variables, between the case and control groups. A P value < 0.05 was considered statistically significant.

## 4. Results

**Table 3.** Comparison of Demographic and Clinical Characteristics Between the Case and Control Groups

Variables	Mean	Standard Deviation	Mean Difference	Standard Error Difference	P-Value
**Age**			0.5	0.32	0.12
Case	9.61	1.88			
Control	9.11	1.82			
**Systolic blood pressure**			2.73	1.53	0.08
Case	105.42	9.43			
Control	102.69	8.25			
**Diastolic blood pressure**			1.40	1.63	0.39
Case	70.64	8.88			
Control	69.23	9.31			
**Weight**			5.27	1.61	0.002
Case	34.91	10.06			
Control	29.64	7.48			
**Height**			6.52	2.29	0.006
Case	138.92	14.14			
Control	132.40	11.10			
**Body Mass Index**			0.98	0.32	0.002
Case	17.64	1.95			
Control	16.66	1.76			
**Body Mass Index percentile**			8.34	4.11	0.04
Case	61.91	21.85			
Control	53.57	24.10			

**Table 4.** Comparison of Mean Cardiac Function Variables Between the Case and Control Groups

Variables and Group	Mean	Standard Deviation	Mean Difference	Standard Error Difference	P-Value
**Ejection fraction**			1.00	1.21	0.41
Case	68.29	7.48			
Control	67.29	6.68			
**Fractional shortening**			-0.59	1.07	0.58
Case	37.69	5.13			
Control	38.28	6.62			
**E**			2.81	2.46	0.25
Case	82.93	14.02			
Control	80.11	14.27			
**A**			2.40	2.25	0.29
Case	53.85	15.04			
Control	51.45	11.87			
**E/A**			0.02	0.06	0.74
Case	1.62	0.37			
Control	1.60	0.24			
**Tei Index**			-0.003	0.01	0.68
Case	0.38	0.06			
Control	0.38	0.05			
**E/E'**			-0.07	0.17	0.68
Case	3.90	1.09			
Control	3.97	0.90			
**E'**			1.57	0.83	0.06
Case	22.08	5.64			
Control	20.50	4.30			
**A'**			1.00	0.43	0.02
Case	11.42	2.83			
Control	10.42	2.31			
**E'/A'**			-0.02	0.14	0.89
Case	2.10	0.77			
Control	2.12	0.85			
**Deceleration time**			3.72	4.15	0.37
Case	105.18	23.30			
Control	101.46	24.15			

^a^ Abbreviations: A, late diastolic peak velocity; A', late diastolic tissue velocity; DT, deceleration time; E, early diastolic filling velocity; E', early diastolic annular peak velocity; TAPSE, tricuspid annular plane systolic excursion.

**Table 5.** Number and Percentage of Sex, Electrocardiographic Changes, Ejection Fraction Status, and Fractional Shortening in the Case and Control Groups Among Patients Referred to the Clinic of Amirkola Children's Hospital ^a^

Variables and Category	Case	Control	P-Value
**Gender**			0.60 ^b^
Boys	24 (48)	54 (54)	
Girls	26 (52)	46 (46)	
**Ejection fraction**			0.77 ^c^
Normal	49 (98)	97 (97)	
Mild impairment	1 (2)	2 (2)	
Moderate impairment	0 (0)	1 (1)	
Severe impairment	0 (0)	0 (0)	
**Fractional shortening**			-
Normal	50 (100)	100 (100)	
Mild impairment	0 (0)	0 (0)	
Moderate impairment	0 (0)	0 (0)	
Severe impairment	0 (0)	0 (0)	

^a^ Values are expressed as No. (%).

^b^ Chi-square test.

^c^ Fisher exact test.

### 4.1. Comparison of Baseline Characteristics

The case group (T_1_DM, n = 50) and control group (n = 100) were comparable in terms of age (P = 0.12), systolic blood pressure (P = 0.08), and diastolic blood pressure (P = 0.39). However, the T_1_DM group had significantly higher mean values for weight (P = 0.002), height (P = 0.006), BMI (P = 0.002), and BMI percentile (P = 0.04). These findings suggest that, in this cohort, children with T_1_DM generally had greater anthropometric measurements than their healthy counterparts, independent of diabetes status.

### 4.2. Global Cardiac Function Parameters

Overall left ventricular systolic function, assessed by EF (P = 0.41) and FS (P = 0.58), did not differ significantly between the T_1_DM and control groups. Similarly, most standard diastolic filling parameters, including the E/A ratio (P = 0.74), the Tei index (P = 0.68), and the E/E' ratio (P = 0.68), showed no significant intergroup differences. However, one key tissue Doppler index, A', was significantly higher in the T_1_DM group (P = 0.02), suggesting a preserved or possibly enhanced atrial contribution to ventricular filling in this cohort, despite overall normal systolic indices.

### 4.3. Incidence of Abnormal Cardiac Findings

The distribution of demographic variables and overall structural measurements, including sex (P = 0.60), EF impairment (P = 0.77), and FS status, did not differ significantly between the case and control groups. Specifically, the incidence of mild to moderate EF impairment was very low in both groups. Furthermore, standard ECG parameters, including heart rate, PR interval, QRS duration, QTc, axis, ST-T changes, and arrhythmias, were comparable between groups (P > 0.05).

### 4.4. Cardiac Function Based on Glycemic Control

When patients were compared according to glycemic control (HbA1c < 7.5% vs ≥ 7.5%), mean EF in the poor control group (66.70 ± 7.84) was numerically lower than that in the good control group (70.90 ± 6.19), reaching marginal statistical significance (P = 0.053). Importantly, other indices of ventricular function, including FS, E/A, TAPSE, the Tei index, E', A', and E/E', showed no significant differences between the good and poor control subgroups (P > 0.05 for all).

### 4.5. Correlation Analysis and Specific Diastolic Impairment

No significant correlation was found between EF and HbA1c or duration of diabetes (r = 0.15, P = 0.29).

A significant difference emerged in the categorical distribution of a reduced E/A ratio, suggesting impaired relaxation. A significantly higher proportion of patients with T_1_DM had a reduced E/A ratio than controls (P = 0.007; 16% in the case group vs 3% in the control group). Furthermore, 2 patients with an elevated Tei index were identified exclusively in the T_1_DM group. Notably, the E'/A' ratio remained normal in all participants.

Within the T_1_DM group, impairment in the E/A ratio was not associated with differences in mean HbA1c level, age, or duration of diabetes (Table 6; all P > 0.05).

**Table 6.** Differences in Mean HbA1c, Age, and Duration of Diabetes According to E/A Ratio Impairment in Children With Type 1 Diabetes Mellitus ^a^

Variables	Impairment in E/A Ratio, No	Impairment in E/A Ratio, Yes	P-Value
**Age**	9.08 ± 1.86	8.41 ± 1.38	0.60
**Duration of diabetes**	4.39 ± 1.67	5.25 ± 2.24	0.21
**HbA1c**	9.55 ± 1.91	9.94 ± 1.78	0.34

^a^ Values are expressed as mean ± SD.

### 4.6. Predictors of Type 1 Diabetes Mellitus Membership

The multivariate logistic regression model identified diastolic indices as the strongest independent predictors of case group membership (Table 7). The E/E' ratio (OR = 4.67, P = 0.032) and E' velocity (OR = 1.33, P = 0.029) were significant predictors. Specifically, each unit increase in E/E' increased the odds of being in the T_1_DM group by approximately 4.7-fold. In contrast, demographic factors, including age, sex, BMI percentile, and systolic function measures (EF and FS), did not significantly contribute to the predictive power of the model.

**Table 7.** Summary of Logistic Regression Results for Factors Predicting Case Group Membership ^a^

Variables	B	Significance	OR (Exp[B])	OR (95% CI)
**E/E'**	1.542	0.032	4.673	1.144 - 19.086
**E'**	0.282	0.029	1.326	1.030 - 1.708
**Age**	0.139	0.278	1.149	0.894 - 1.477
**Gender**	0.372	0.413	1.451	0.595 - 3.541
**Systolic blood pressure**	0.056	0.094	1.058	0.991 - 1.129
**Diastolic blood pressure**	-0.027	0.366	0.973	0.918 - 1.032
**Body mass index percentile**	0.014	0.129	1.014	0.996 - 1.033
**Ejection fraction**	0.060	0.358	1.062	0.934 - 1.208
**Fractional shortening**	-0.054	0.568	0.947	0.786 - 1.141
**E**	-0.083	0.068	0.920	0.842 - 1.006
**A**	0.027	0.497	1.027	0.950 - 1.111
**E/A**	1.442	0.371	4.231	0.179 - 99.888
**TAPSE**	-0.081	0.261	0.922	0.800 - 1.062
**A'**	0.230	0.099	1.258	0.958 - 1.653
**E/A'**	0.132	0.773	1.141	0.465 - 2.796

^a^ Abbreviations: E/E’: Early mitral inflow to early diastolic annulus velocity ratio; E’: early diastolic mitral annular velocity; SBP: systolic blood pressure; DBP: diastolic blood pressure; BMI: Body Mass Index; EF: ejection fraction; FS: fractional shortening; E: peak early diastolic mitral inflow velocity; A: peak late diastolic mitral inflow velocity; E/A: ratio of peak early to late diastolic mitral inflow velocities; TAPSE: tricuspid annular plane systolic excursion; A’: late diastolic mitral annular velocity; E/A’: ratio of early to late diastolic mitral annular velocities.

## 5. Discussion

The present study evaluated cardiac function in children aged 6 - 12 years with T_1_DM and compared them with healthy children. The findings indicated that the mean values of most cardiac indices, including EF, FS, E, A, E/A, TAPSE, Tei index, E/E', E', E'/A', and DT, were similar between the diabetic and healthy groups, whereas the mean value of A' was elevated in the diabetic group. In addition, the frequency of an increased E/A ratio was higher in the case group. Two children with an elevated Tei index also belonged to the case group. These results underscore the high prevalence of diastolic dysfunction in pediatric T_1_DM. The observed changes, particularly the statistically significant elevation in mean A' velocity, place our findings within the context of emerging evidence of early functional impairment and suggest that diastolic alterations may manifest before, or concomitantly with, detectable systolic compromise in this population.

The elevation in mean A' velocity likely reflects an early adaptive or hypercontractile phase of subclinical diastolic dysfunction, supporting its consideration as a novel, sensitive biomarker for identifying high-risk pediatric patients with T_1_DM who may warrant more intensive early cardiological management.

These findings are partially consistent with other studies regarding global systolic function, including EF and FS, and align with reports by Rakha et al. (40), Ozdemir et al. (41), Aziz et al. (42), and Zairi et al. (43). However, a notable discrepancy exists regarding diastolic parameters. While Rakha et al. (40) reported a significantly higher E/E' ratio in their T_1_DM cohort, the present study found no significant abnormality in the E/E' ratio. This directly contrasts with their findings but is more consistent with the study by Ozdemir et al. (41), who observed impairments in other indices, such as the E/A ratio. Importantly, our observation of elevated mean A' velocity, while contrasting with the normative findings of Rakha et al. (40) in Egypt, suggests that our cohort may represent a distinct and potentially earlier stage of diastolic compromise characterized by compensatory hyperkinesis rather than the restrictive filling pattern suggested by an elevated E/E'. The isolated elevation of A' observed in this study, in the context of preserved E', a normal E/E' ratio, and normal systolic indices, likely represents an early adaptive phase of diastolic dysfunction characterized by increased atrial contribution to ventricular filling rather than established myocardial stiffness or elevated filling pressures. This pattern has been described in pediatric populations as a precursor stage preceding overt diabetic cardiomyopathy (23).

Although the present study suggested that mean EF was higher in diabetic children with good glycemic control than in those with poor control, this difference was not significant. Diabetes duration was also not significantly associated with EF. This contrasts with the study by Adoe et al. (44) in Indonesia, which indicated a slight significant association between left ventricular diastolic function and HbA1c. However, Hussein et al. in Iraq and Caglar Acar et al. (45) in Turkey also found no differences in mean EF across HbA1c levels and no association with diabetes duration, which agrees with the current study. The lack of statistical significance in EF differences may be because EF alone cannot fully capture complex myocardial deformation, limiting its accuracy in assessing systolic function (43).

A notable aspect of the study was the assessment of differences in mean HbA1c, age, and diabetes duration according to E/A ratio impairment, with no differences observed. Based on these results, we recommend that children with T_1_DM receive regular cardiac monitoring for at least 3 years after diagnosis. Future investigations should include long-term follow-up and alternative methods, such as carotid ultrasound, to determine cardiovascular disease risk.

The strengths of this study include the achieved patient enrollment (n = 50 cases) and the 2:1 allocation ratio for controls (n = 100), which maximized statistical power given the case sample size.

This study has several important methodological constraints that warrant careful interpretation. First, its cross-sectional design limits causal inference regarding the relationship between T_1_DM and cardiac function. Second, the relatively small case sample size precluded full statistical adjustment for potential confounders, such as BMI, age, sex, and diabetes duration. Consequently, residual confounding is likely. Third, the use of convenience sampling may introduce selection bias, as participants from a single tertiary clinic may not represent the broader population of children with diabetes. Although all participants were prepubertal and within the normal BMI range, minor anthropometric differences in height, weight, and BMI could still influence diastolic indices.

Despite these limitations, the study benefited from a standardized, single-center setting, which allowed high consistency in echocardiographic measurements, a methodological strength that enhances internal validity. Nonetheless, future multicenter longitudinal investigations with larger and z score-adjusted samples are recommended to confirm and expand the external validity of our findings.

### 5.1. Conclusion

This preliminary cross-sectional study found a higher frequency of indices of diastolic impairment in children with T_1_DM than in healthy controls. These observations suggest a potential association, indicating that T_1_DM may be linked to early alterations in cardiac function during the initial years of the disease. These findings warrant further investigation to establish causality and validate A' as a biomarker in larger longitudinal cohorts.

## Data Availability

The dataset presented in the study is available on request from the corresponding author during submission or after publication.
